# Comprehensive analysis of the prognostic value and functions of prefoldins in hepatocellular carcinoma

**DOI:** 10.3389/fmolb.2022.957001

**Published:** 2022-11-11

**Authors:** Shanjia Ke, Shounan Lu, Chaoqun Wang, Yanan Xu, Miaoyu Bai, Hongjun Yu, Zhigang Feng, Bing Yin, Zihao Li, Jingjing Huang, Xinglong Li, Baolin Qian, Yongliang Hua, Shangha Pan, Yaohua Wu, Yong Ma

**Affiliations:** ^1^ Department of Minimal Invasive Hepatic Surgery, The First Affiliated Hospital of Harbin Medical University, Harbin, China; ^2^ Key Laboratory of Hepatosplenic Surgery, Ministry of Education, The First Affiliated Hospital of Harbin Medical University, Harbin, China; ^3^ The First Department of General Surgery, Affiliated Hospital of Inner Mongolia Minzu University, Tongliao, Inner Mongolia, China; ^4^ Department of Pediatric Surgery, The First Affiliated Hospital of Harbin Medical University, Harbin, China; ^5^ Department of Thyroid Surgery, The First Affiliated Hospital of Harbin Medical University, Harbin, China

**Keywords:** bioinformatics analysis, PFDNs, hepatocellular carcinoma, biomarkers, prognosis

## Abstract

Prefoldins (PFDNs), a group of proteins known to be associated with cytoskeletal rearrangement, are involved in tumor progression in various cancer types. However, little is known about the roles of PFDNs in hepatocellular carcinoma (HCC). Herein, we investigated the transcriptional and survival data of PFDNs from The Cancer Genome Atlas (TCGA) database. Gene Ontology (GO), Gene Set Enrichment Analysis (GSEA), and single-sample gene set enrichment analysis (ssGSEA) were used to evaluate the potential functions of PFDN1/2/3/4. We also detected the expression of PFDN1/2/3/4 *via* immunohistochemistry (IHC), Western blotting, and real-time PCR in our clinical samples. We found that the PFDN family showed elevated expression in HCC tissues, while only PFDN1/2/3/4 were found to be significantly correlated with poor prognosis of patients with HCC in the TCGA database. Further investigation was associated with PFDN1–4. We found that the expression of PFDN1/2/3/4 was significantly associated with advanced clinicopathologic features. Apart from the TCGA database, IHC, real-time PCR, and immunoblotting identified the overexpression of PFDN1/2/3/4 in HCC tissues and HCC cell lines. Taken together, these results indicated that PFDN1/2/3/4 might be novel prognostic biomarkers and treatment targets for patients with HCC.

## Introduction

Primary liver cancer is the sixth most common malignant tumor and ranks fourth in cancer-related death worldwide (Villanueva, 2019). More than 1 million patients are estimated to die from liver cancer in 2030, based on annual projections from the World Health Organization. Hepatocellular carcinoma (HCC) is the most common type of liver cancer because of hepatitis virus (HBV and HCV) infection, alcohol abuse, or non-alcoholic fatty liver disease (NAFLD) (Geier et al., 2002; Craig et al., 2020). Although significant progressions in treatment methods have been achieved, HCC patients who underwent liver surgery are more likely to suffer from postoperative recurrence and distant metastasis, and their 5-year overall survival is less than 20% (Ji and Wang, 2012; Llovet et al., 2015). In addition, due to lack of sensitive diagnostic markers, patients with HCC are more likely to be diagnosed at advanced stages and lose treatment opportunities. Thus, we must improve the diagnosis and treatment of patients with liver cancer by identifying novel biomarkers and exploring the molecular mechanisms of HCC.

Prefoldins (PFDNs), which contain six different subunits (PFDN1–6), are a jellyfish-like co-chaperone promoting correct protein synthesis in the cytosol, especially actin and tubulin monomers (Vainberg et al., 1998). PFDNs in chaperonin-mediated folding are not only specific to cytoskeleton components but are also involved in assembling cytoplasmic complexes and maintaining the stability of functional proteins. In addition to these functions, members of the PFDN family also participate in regulating the transcription of genes in the nucleus, including removal of HIV integrase and repression of c-Myc (Mori et al., 1998; Boulon et al., 2012; Payán-Bravo et al., 2018; Esteve-Bruna et al., 2020). A recent study reported that the family of prefoldins regulates co-transcriptional pre-mRNA splicing by supporting the recruitment of the splicing machinery to the human genome during transcript elongation (Payán-Bravo et al., 2021).

Therefore, the dysregulated expression of PFDN may lead to various diseases (Mo et al., 2020). Every subunit of PFDN has different functions, and their roles in cancer seem contradictory. For example, PFDN1 promotes EMT and lung cancer development by suppressing the expression of cyclin A mRNA and enhances colorectal cancer progression *via* cytoskeletal reorganization (Wang et al., 2015; Wang et al., 2017). PFDN2 disturbs the organization of the tubulin cytoskeleton by interacting with hepatitis C virus F protein and predicts a poor prognosis for patients with gastric cancer (Tsao et al., 2006; Yesseyeva et al., 2020). PFDN3, also known as von Hippel–Lindau (VHL)-binding protein 1 (VBP1), represses cancer metastasis by enhancing HIF-1α degradation induced by pVHL (Kim et al., 2018). High PFDN4 expression predicts better prognosis of patients with colorectal cancer (Miyoshi et al., 2010). PFDN5 accelerates cell migration by contributing to filopodium formation and promotes degradation of c-Myc by recruiting an E3 ubiquitin ligase complex (Kimura et al., 2007; Fan et al., 2020). In addition, PFDN6 serves as a potential biomarker of the prognosis of childhood acute lymphoblastic leukemia (ALL) and might predict the efficacy of chemotherapy (Dehghan-Nayeri et al., 2017). PFDN2, PFDN6, and PFD-like proteins (URI, UXT, and PDRG1) form a noncanonical complex that acts as a co-chaperone of Hsp90 (Houry et al., 2018). Nevertheless, potential research on the PFDN family in HCC is still limited.

This study aimed to investigate the prognostic value and potential function of the PFDN family in HCC using gene sequencing technology and bioinformatics analysis. Based on clinicopathological parameters and survival information from The Cancer Genome Atlas (TCGA), we comprehensively analyzed the relationship between the six PFDN members and HCC. Clinical samples from patients with HCC were subsequently used to analyze the differential expression of PFDN1-4.

## Materials and methods

### Data and preprocessing

The mRNA expression data and clinicopathological information for patients with HCC (containing 374 tumors and 50 normal tissues) were downloaded from TCGA (https://portal.dgc.cancer.gov/). Exclusion criteria were OS less than 30 days and normal HCC tissues. Then, the FPKM information of 374 HCC samples was further analyzed. Unknown and unavailable clinical parameters from 374 samples were considered missing values, and the information is shown in Supplementary Table S1.

### Expression of the PFDN family in HCC samples from the Oncomine and TCGA datasets

Oncomine datasets (https://www.oncomine.org/resource/login.html) were used to analyze the PFDN mRNA expression levels in different cancers. The transcriptional expression of PFDNs in tumor specimens was compared with those in normal tissues or adjacent normal tissues in the TCGA database, using Wilcoxon rank sum tests or Wilcoxon signed-rank tests. Using the pROC (1.17.01) package, a receiver operating characteristic (ROC) curve was constructed to evaluate the performance of the PFDN family in discriminating patients with HCC. Based on statistical ranking, PFDN1 expression greater or less than the median was recognized as the PFDN1-high or PFDN1-low group. Applying the DEseq2 (4.0) package and Student’s t test, differentially expressed genes (DEGs) from TCGA between the PFDN1-low and PFDN1-high groups were analyzed, using an absolute log(FC) greater than 1.5 and adj *p-value* < 0.05. The other three genes (PFDN2-4) were analyzed using similar methods. The DEGs of PFDN1/2/3/4 are shown in Supplementary Table S2–S5.

### Clinical analysis of the prognosis and pathological parameters associated with PFDN1-4

The survival curve was generated using the survival R package (3.2–10) and the survminer R package (0.4.9). The relationships between clinicopathological characteristics and PFDN1–4 expression were determined using the Kruskal–Wallis test or Wilcoxon signed-rank test and logistic regression analysis.

### Analysis of immune cell infiltration

Based on the TCGA database, we performed single-sample gene set enrichment analysis (ssGSEA) from “GSVA” (R package) to quantify the relative infiltration of 24 types of immunocytes (Bindea et al., 2013; Hänzelmann et al., 2013). Spearman’s correlation and Wilcoxon rank sum tests were used to investigate the correlations between PFDN1-4 expression and different immune cells, as well as the association of infiltration of Th2 and Tfh cells with high- and low-expressing PFDN (PFDN1–4) groups.

### Construction of the PPI network and gene set enrichment analysis

A PFDN1–4-related protein–protein interaction (PPI) network was constructed using the Search Tool for the Retrieval of Interacting Genes/Proteins (STRING) database (https://string-db.org/) (Szklarczyk et al., 2019) to explore the connections between PFDN1–4 and other genes. A minimum required interaction score >0.7 and Cytoscape 3.7.1 (Shannon et al., 2003) were applied to visualize these interactions and further identify the key genes related to PFDN1-4. In addition, Gene Ontology (GO) and Kyoto Encyclopedia of Genes and Genome (KEGG) analyses were performed on key genes with the package R “clusterProfiler” to identify potential biological functions affected by PFDN1–4 (Yu et al., 2012). In this study, GSEA was performed using the Molecular Signatures Database (MSigDB). The differences in biofunctions between the PFDN1-low and PFDN1-high group were analyzed by the R package clusterProfiler. An adjust *p-value* < 0.05 and false discovery rate (FDR) q-value<0.25 were deemed criteria to identify significantly associated biological pathways. The other three genes (PFDN2–4) were analyzed using similar methods.

### Construction and evaluation of the nomogram and prognostic model

Based on a multivariate Cox regression analysis, a nomogram was constructed to predict 1-year, 3-year, and 5-year survival probabilities. We used the rms R package to construct the nomogram. The Hmisc R package was used to evaluate the C-index and calibration plot. In this study, we applied the C-index to determine the discrimination ability with 1000 bootstrap resamples.

### Hepatocellular carcinoma specimens and hepatocellular carcinoma cells

We collected paired HCC and adjacent noncancerous tissue from patients who underwent surgical resection at the First Affiliated Hospital of Harbin Medical University between May 2020 and May 2021. Ethical approval was obtained from The First Affiliated Hospital of Harbin Medical University Research Ethics Committee, and informed consent was obtained from each patient. The human HCC cell lines HepG2, Huh7, SK-Hep-1, HCCLM3, and Bel-7402 were obtained from the Chinese Academy of Science (Shanghai, China). Normal WRL-68 liver cells were obtained from AcceGen (Fairfield, United States). All cell lines were cultured in Dulbecco’s modified Eagle’s medium (Gibco, United States) supplemented with 10% fetal bovine serum (Gibco, United States), 100 U/mL penicillin, and 100 µg/ml streptomycin. All cells were incubated in incubators containing 5% CO_2_ at 37°C.

### Quantitative real-time PCR and immunoblotting analysis

Quantitative real-time PCR and Western blot analyses were performed as previously described (Wang et al., 2021). Detailed information about the primers used for quantitative real-time PCR is given in Supplementary Table S6. Information regarding all primary antibodies used in this study is given in Supplementary Table S7.

### Immunohistochemical staining

After a series of processes including dewaxing, rehydration in a graded series of alcohol solutions, antigen retrieval, and blocking with goat serum, tissue sections were incubated with primary antibodies overnight. The following day, tissue sections were incubated with secondary antibodies (Vector Laboratories, United States) and stained with diaminobenzidine (Vector Laboratories). The protein staining intensity score was calculated using previously described methods (Wang et al., 2021).

### Statistical analysis

All statistical analyses were conducted with R (version 3.6.3). One-way analysis of variance (ANOVA) and two tailed Student’s t test were used to analyze the data. All figures were plotted using the R package ggplot2 (3.3.3). *p*-value of< 0.05 was considered statistically significant.

## Results

### Increased expression of the PFDN family in hepatocellular carcinoma patients

**FIGURE 1:**
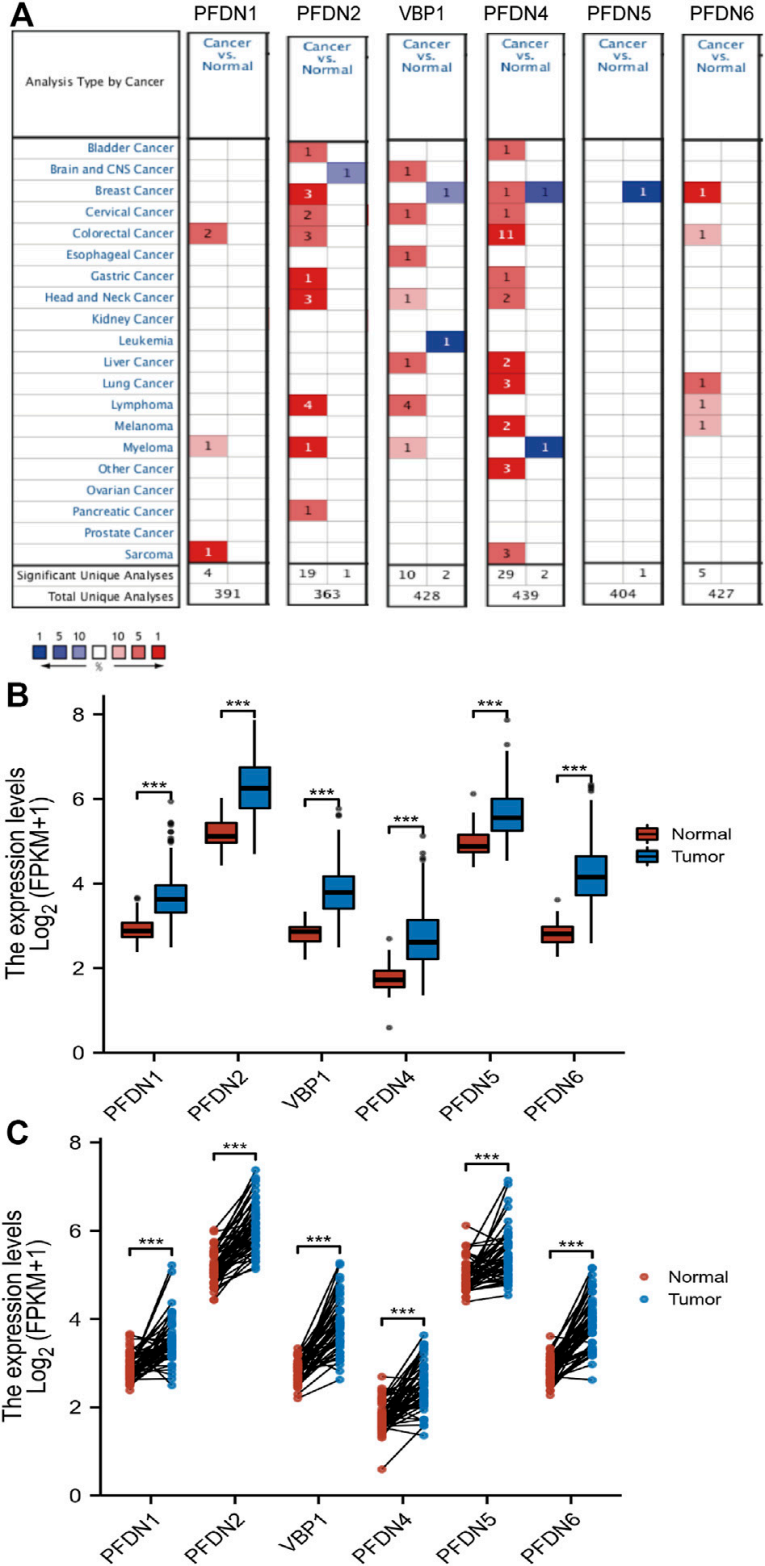
Differential expression levels of the PFDN family. **(A)** Transcription levels of the PFDN family in different cancers (Oncomine). **(B,C)** Expression levels of the PFDN family in HCC.

First, the Oncomine database was chosen to compare the mRNA levels of the PFDN family between tumor and normal tissues. The results showed increased expression of the PFDN family in multiple cancer tissues (Figure 1A). The Oncomine analysis also indicated that PFDN3/4 was significantly overexpressed in HCC tissues, with fold changes of 2.364 and 2.758 and *p-value* of 4.76E-66 and 6.50E-71, in the Roessler Liver 2 dataset (Roessler et al., 2010). In the Roessler Liver dataset, PFDN4 was also overexpressed in HCC patients, with a fold change of 3.253 and a *p-value* of 6.11E-11 (30). In addition, an analysis of the TCGA database showed significantly higher mRNA levels of the PFDN family in HCC tissues than in liver tissues or adjacent liver tissues (Figures 1B,C). In the TCGA database, we found 41 paired clinical samples show increased expression of PFDN1 (41/51); 49 paired clinical samples show increased expression of PFDN2 (49/51); 49 paired clinical samples show increased expression of VBP1 (49/51); 43 paired clinical samples show increased expression of PFDN4 (43/51); 42 paired clinical samples show increased expression of PFDN5; 49 paired clinical samples show increased expression of PFDN6 in HCC tissues. The AUCs for the PFDN family were 0.916, 0.926, 0.952, 0.904, 0.850, and 0.971, respectively (Supplementary Figure S1). This result suggested that these genes had potential value to discriminate HCC and normal liver tissues.

### Prognostic value of the level of PFDN transcripts in HCC

**FIGURE 2:**
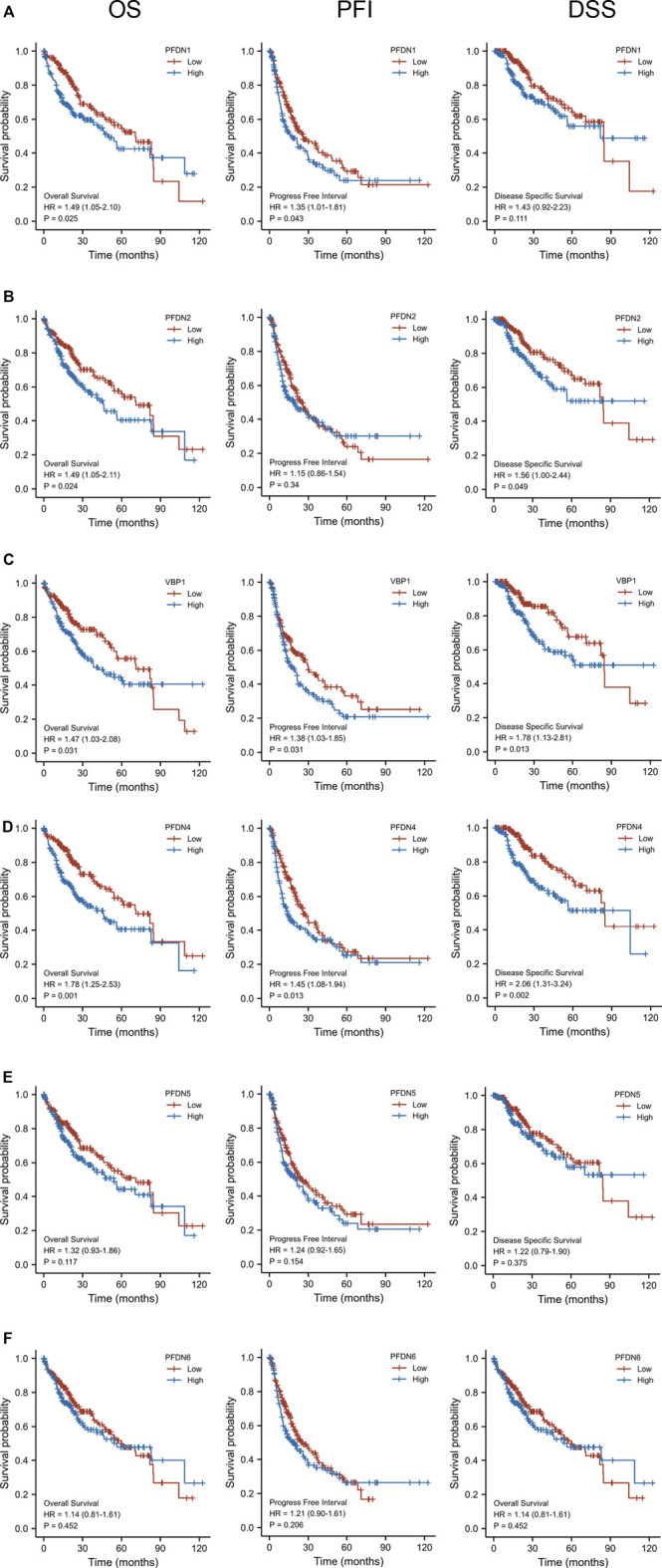
Prognostic value of the mRNA level of the PFDN family in HCC (Kaplan–Meier Plotter). **(A)** Higher expression of PFDN1 was significantly associated with OS and PFI of patients with HCC. **(B)** Higher transcriptional expression of PFDN2 was significantly associated with OS and DSS of patients with HCC. **(D,E)** Higher expression of VBP1 and PFDN4 was significantly associated with OS, PFI, and DSS of patients with HCC. **(F,G)** However, PFDN5 and PFDN6 mRNA expression showed no correlations with prognosis of patients with HCC.(a)(b)

After identifying the differential expression of the PFDN family in HCC, we subsequently explored the relationship between the transcriptional expression of the PFDNs and the clinical outcomes of patients with HCC using the TCGA database. Overall survival (OS), progression-free interval (PFI), and disease-specific survival (DSS) are presented in Figure 2. These results indicated that, with the exception of PFDN5 and PFDN6, higher expression of PFDN1–4 was significantly correlated with worse clinical outcomes of patients with HCC. Higher expression of PFDN1 (HR = 1.49, 95% CI: 1.05–2.10, *p* = 0.025), PFDN2 (HR = 1.49, 95% CI: 1.05–2.11, *p* = 0.024), VBP1 (HR = 1.47, 95% CI: 1.03–2.08, *p* = 0.031), and PFDN4 (HR = 1.78, 95% CI: 1.25–2.53, *p* = 0.001) was significantly associated with shorter OS of liver cancer patients. HCC patients expressing higher levels of the PFDN1 (HR = 1.35, 95% CI: 1.01–1.81, *p* = 0.043), VBP1 (HR = 1.38, 95% CI: 1.03–1.85, *p* = 0.031), and PFDN4 (HR = 1.45, 95% CI: 1.08–1.94, *p* = 0.013) transcripts experienced significantly shorter PFI. In addition, increased expression of PFDN2 (HR = 1.56, 95% CI: 1.00–2.44, *p* = 0.049), VBP1 (HR = 1.78, 95% CI: 1.13–2.81, *p* = 0.013), and PFDN4 (HR = 2.06, 95% CI: 1.31–3.24, *p* = 0.002) was significantly correlated with a shorter DSS. Based on these findings, PFDN1/2/3/4 were significantly associated with prognosis of patients with HCC, and these genes become useful biomarkers to predict the survival of liver cancer patients.

### Correlations between the expression of the PFDN1/2/3/4 mRNAs and clinicopathological variables

**FIGURE 3:**
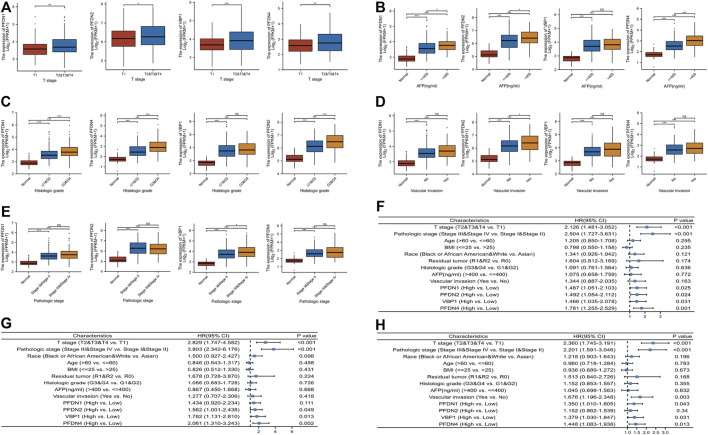
Relationship between mRNA expression of PFDN1/2/3/4 and clinical parameters of patients with HCC. The correlation between PFDN1/2/3/4 and **(A)** T stage, **(B)** AFP level, **(C)** histologic grade, **(D)** vascular invasion, and **(E)** pathologic stage. **(F)** PFDN1/2/3/4 has a prominent effect on OS. **(G)** PFDN2/3/4 has a prominent effect on DSS. **(H)** PFDN1/3/4 has a prominent effect on PFI.

In addition to the prognostic value of PFDN1–4, we intended to investigate the relationship between the levels of PFDN1–4 transcripts and the clinical parameters of patients with HCC. Figures 3A–E show that higher expression of the PFDN1 and PFDN4 mRNAs was significantly related to the T stage (T2 &T3 &T4 *vs*. T1, *p* = 0.002; *p* = 0.009), AFP (>400 *vs*. <400, *p* = 0.007; *p* < 0.001), and histological grade (G3 &G4 *vs*. G1 &G2, *p* < 0.001; *p* < 0.001). Increased expression of PFDN2 was significantly correlated with the T stage (T2 &T3 &T4 *vs*. T1, *p* = 0.037), AFP level (>400 *vs*. <400, *p* = 0.014), histological grade (G3 &G4 *vs*. G1 &G2, *p* < 0.001), and vascular invasion (yes *vs*. no, *p* = 0.005). VBP1 expression was significantly higher in HCC patients with T2 &T3 &T4 stage than in T1 stage (*p* < 0.001) and higher in patients with HCC of pathological grade Ⅲ &Ⅳ than in stage Ⅰ &Ⅱ HCC patients (*p* = 0.009). Moreover, we also performed univariate Cox regression of OS, DSS, and PFI of HCC patients with PFDN1-4 (Figure 3F–H). The results disclosed that, apart from clinical factors, poorer OS was prominently associated with PFDN1 expression (high *vs*. low; *p* = 0.025, HR = 1.487 (95% CI: 1.051–2.103)), PFDN2 expression (high *vs*. low; *p* = 0.024, HR = 1.492 (95% CI: 1.054–2.112)), VBP1 expression (high *vs*. low; *p* = 0.031, HR = 1.466 (95% CI: 1.035–2.078)), and PFDN4 expression (high *vs*. low; *p* = 0.001, HR = 1.781 (95% CI: 1.255–2.529)). The expression of PFDN2 (high *vs*. low; *p* = 0.049, HR = 1.437 (95% CI: 1.001–2.438)), VBP1 (high *vs*. low; *p* = 0.013, HR = 1.782 (95% CI: 1.131–2.810)), and PFDN4 (high *vs*. low; *p* = 0.001, HR = 2.061 (95% CI: 1.310–3.243)) exerted prominent effects on DSS. In addition, a shorter PFI of patients with HCC was prominently correlated with PFDN1 expression (high *vs*. low; *p* = 0.043, HR = 1.350 (95% CI: 1.010–1.805)), VBP1 expression (high *vs*. low; *p* = 0.031, HR = 1.379 (95% CI: 1.030–1.847)), and PFDN4 expression (high *vs*. low; *p* = 0.013, HR = 1.448 (95% CI: 1.083–1.938)). These results suggested that PFDN1-4 might indicate worse clinical outcomes of patients with HCC.

### Connections between the expression of PFDN1-4 and immune cell infiltration

**FIGURE 4:**
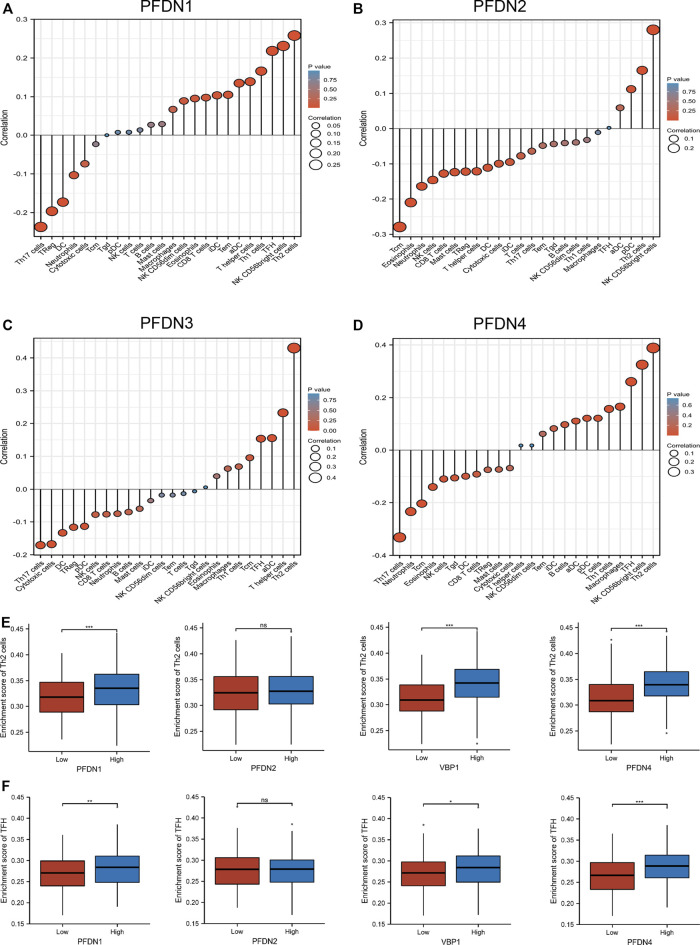
Expression of PFDN1/2/3/4 was related to immune infiltration of the tumor microenvironment. **(A–D)** Relationships between PFDN1/2/3/4 expression and relative abundances of 24 immune cells. **(E,F)** Differential enrichment of TFH and Th2 cells in PFDN1-/2-/3-/4-high groups compared with PFDN1-/2-/3-/4-low groups, respectively.(a)(b)

Infiltrating immune cells undertake crucial roles in the tumorigenesis and metastasis of HCC. According to previous studies, Th2 and Tfh cells contributed to tumor progression and metastasis by suppressing immune system function (De Monte et al., 2011; Fleming et al., 2017; Crotty, 2019). In the present study, an ssGSEA of the HCC tumor microenvironment, and Spearman’s correlation analysis were performed to evaluate the association between immune cell infiltration and the expression of four members of the PFDN family (Figures 4A–D). PFDN1–4 expression was positively correlated with Th2 cell infiltration (R = 0.26, *p* < 0.001; R = 0.17, *p* < 0.001; R = 0.43, *p* < 0.001; and R = 0.39, *p* < 0.001, respectively) (Supplementary Figure S2A). The expression of PFDN1/3/4 levels was positively correlated with Tfh cell infiltration (R = 0.218, *p* < 0.001; R = 0.154, *p* = 0.003; and R = 0.26, *p* < 0.001, respectively) (Supplementary Figure S2B). In addition, the infiltration of Th2 and Tfh cells was significantly enriched in the PFDN (1/3/4)-high group compared with the PFDN (1/3/4)-low group of patients with HCC (Figures 4E,F). This result implied that PFDN1-4 might promote the progression of HCC by modulating the immune environment.

### Potential functions and pathways regulated by PFDN1–4 alterations in patients with hepatocellular carcinoma

**FIGURE 5:**
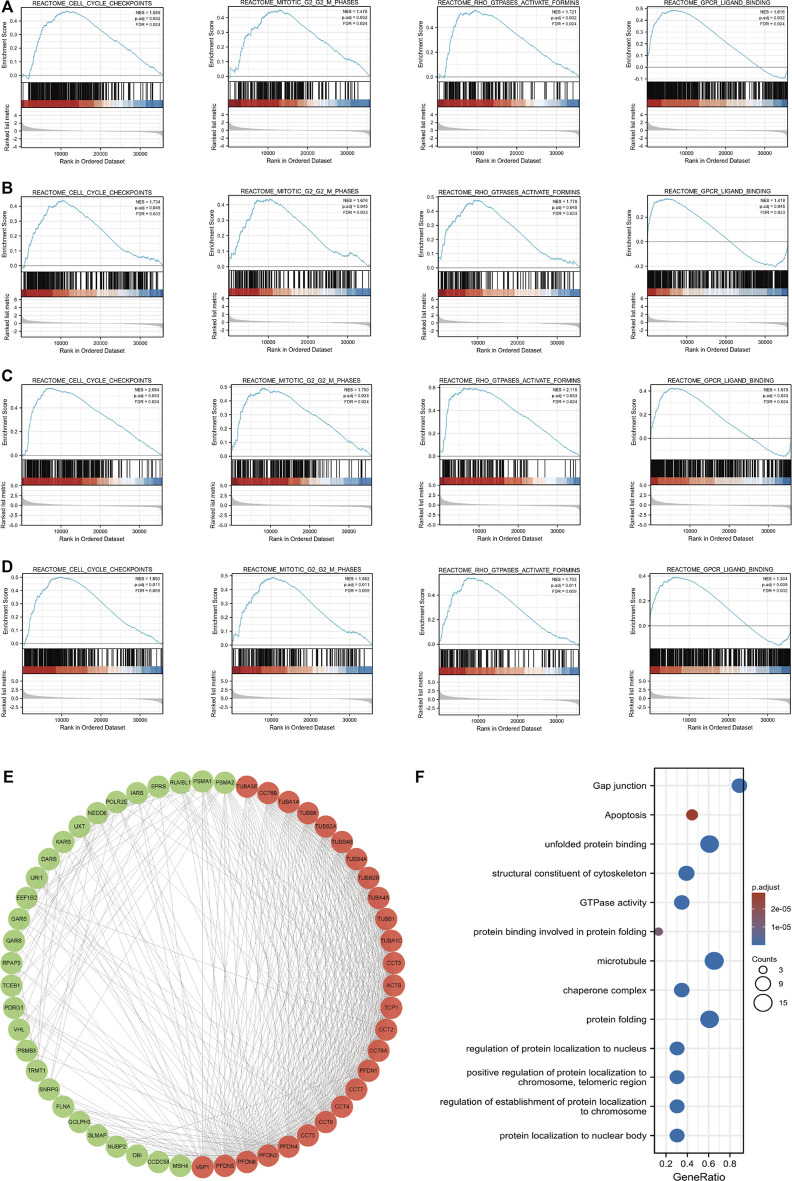
Enrichment plots from GSEA and PPI network of PFDN1/2/3/4. Results of GSEA showed that cell cycle checkpoint, G2-M phases, Rho GTPase activation, and GPCR ligand binding were overlapping functions with PFDN1/2/3/4 alterations. These functions also were significantly enriched in the high-PFDN1 expression group **(A)**, in the high-PFDN2 expression group **(B)**, in the high-PFDN3 expression group **(C)**, and in the high-PFDN4 expression phenotype **(D)**. **(E)** Cytoscape MCODE app selected 50 genes. **(F)** GO analysis of PFDN1-/2-/3-/4-related genes.(a)(b)

GSEA was used to screen significant differences between the low- and high-PFDN1 expression group (adjusted *p* < 0.05, FDR q value < 0.25) in the enrichment of MSigDB Collection (c2.cp.v7.2.symbols.gmt). The other three genes (PFDN2/PFDN3/PFDN4) were analyzed using a similar method (Supplementary Table S8–S11). In addition, we constructed a Venn diagram to find the overlapping part of the GSEA results. Among the intersecting parts, we found that cell cycle checkpoints, mitotic G2-M phases, Rho GTPase activation, and GPCR ligand binding were significantly associated with PFDN1/2/3/4 alterations, and these pathways are also referred to the progression and tumorigenesis of HCC (Figures 5A–D).

### Protein–protein interaction network and analysis of related biofunction

Using the STRING database, we intended to investigate the interactions between PFDN1–4 and other genes. The interaction score was chosen as 0.7. Then, Cytoscape software was used to visualize the protein–protein interactions. Furthermore, MCODE embedded in Cytoscape was used to identify the key gene clusters in the network (Figure 5E). These genes were subjected to GO and KEGG analyses using the R package clusterProfiler (3.14.3) (Supplementary Table S12). Figure 5F shows that the GO functions of these four genes in the PFDN family were mainly related to protein folding, regulation of protein localization in the nucleus and chromosome, and GTPase activity. KEGG analysis showed that gap junctions, apoptosis, and tight junctions were related to tumor progression.

### Construction and evaluation of the nomogram including PFDN1/2/3/4 for patients with hepatocellular carcinoma

**FIGURE 6:**
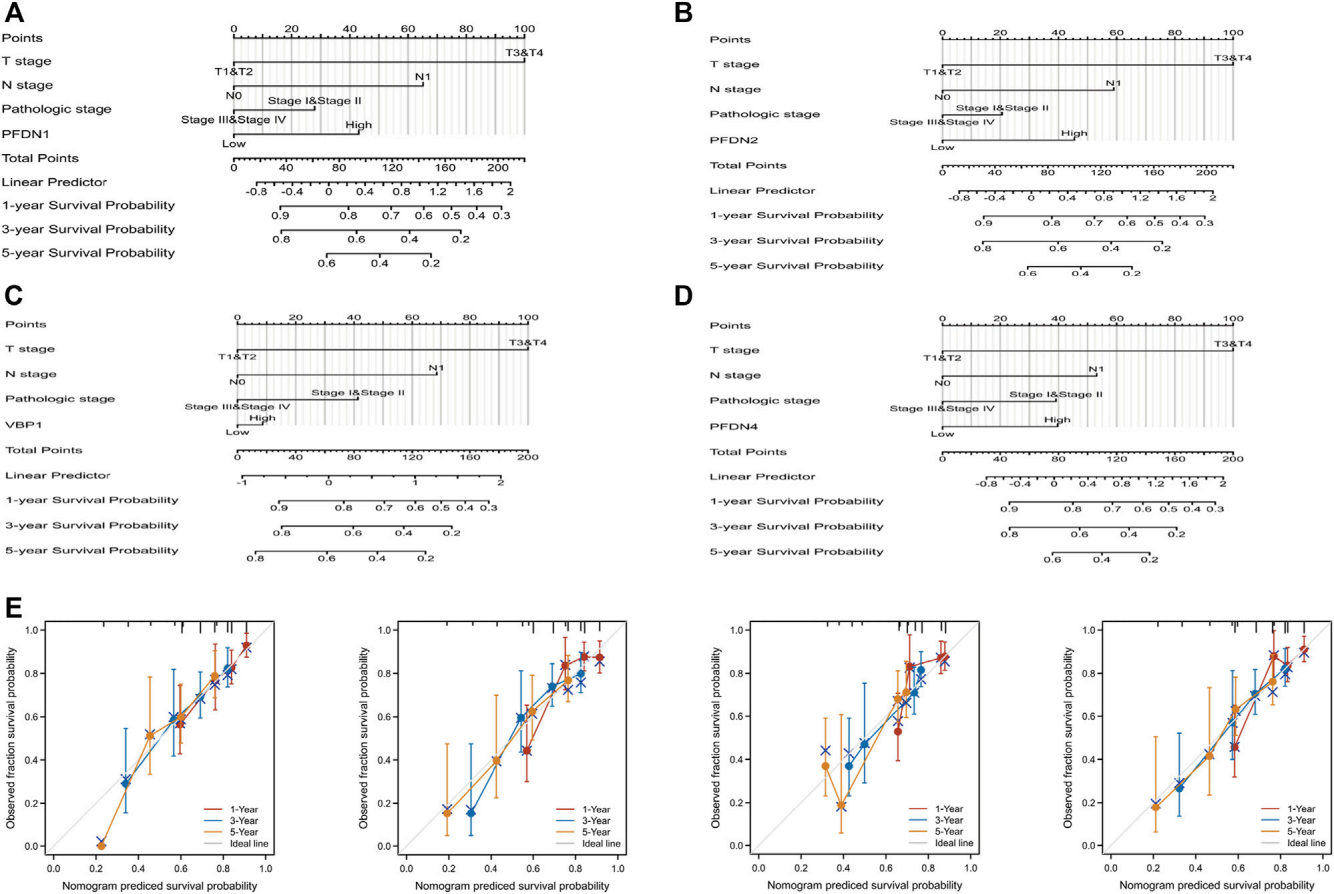
Quantitative method to predict HCC patients’ OS of 1, 3, and 5 years. **(A–D)** Nomograms for estimating the probability of 1-, 3-, and 5-year OS for HCC patients. **(E)** Calibration plots of the nomograms of PFDN1-4 for evaluating the probability of OS at 1, 3, and 5 years.

PFDN1-4 expression and other independent clinicopathological factors were used to construct nomograms that would better predict the survival rates of patients with HCC (Figures 6A–D). Based on a multivariate Cox analysis of the nomogram, a point scale was used to assign points to these variables. The points of the variables were accumulated and recorded as the total scores. A worse prognosis was reflected by higher points in the nomogram. A calibration plot was used to analyze the prediction efficiency of the nomogram (Figures 6E–H). We observed that the C-index of the nomograms was 0.647 (95% CI, 0.618–0.677), 0.634 (95% CI, 0.600–0.669), 0.623 (95% CI, 0.588–0.657), and 0.650 (95% CI, 0.617–0.682). Based on this result, the prognostic nomograms of PFDN1/2/3/4 have relatively good discrimination ability.

### Identification of the differential expression of PFDN1/2/3/4 in our clinical samples

**FIGURE 7:**
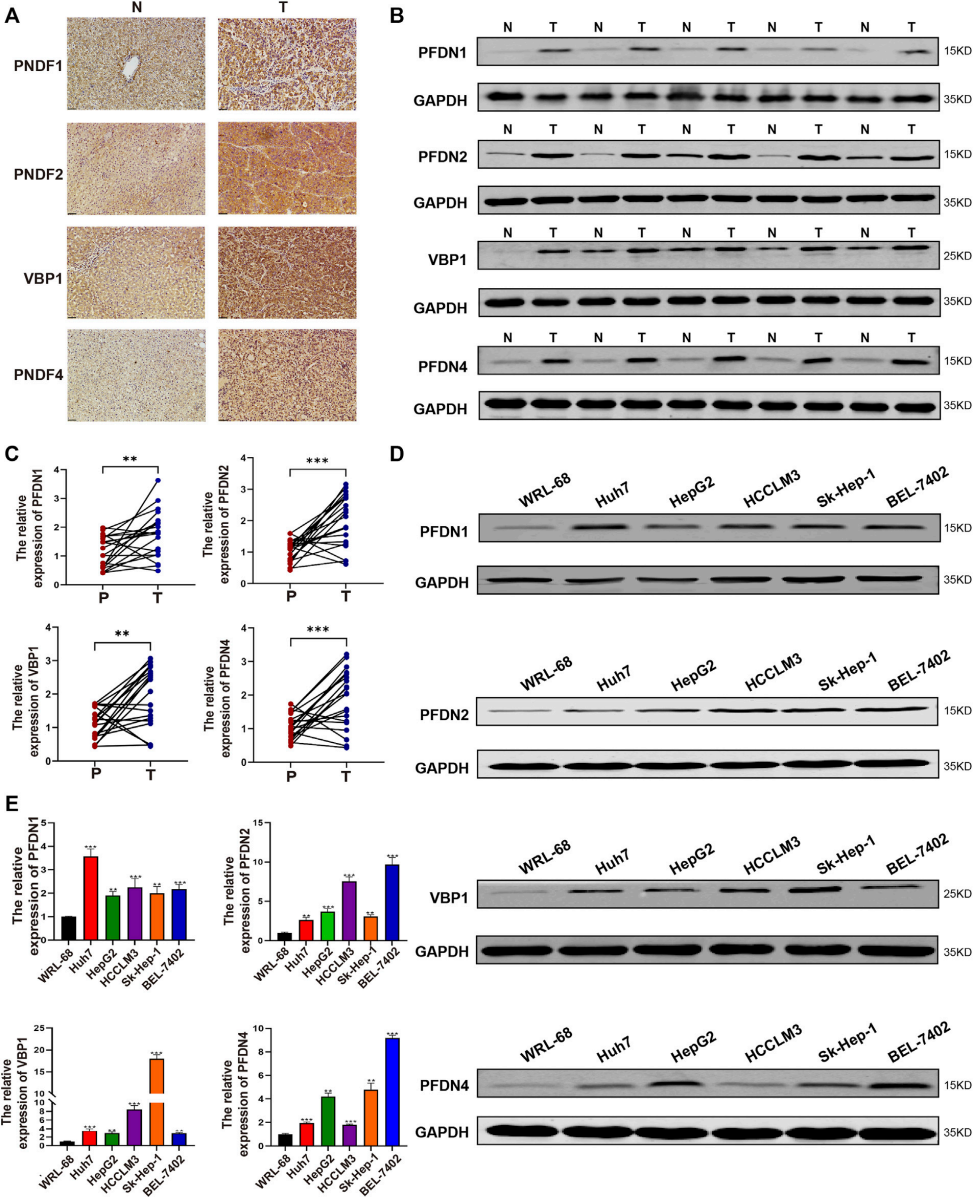
Expression of PFDN1–4 in clinical samples and HCC cell lines. **(A–C)** Expression of PFDN1/2/3/4 in HCC samples (IHC, WB, and real-time qPCR). **(D,E)** Expression of PFDN1/2/3/4 in the normal liver cell line and HCC cell lines (real-time qPCR, WB).

After analyzing the TCGA database using bioinformatics methods, we subsequently determined the differential expression of PFDN1/2/3/4 in our clinical samples and cell lines. PFDN1/2/3/4 was substantially overexpressed in HCC tissues compared with adjacent noncancerous tissues, as determined by real-time PCR (Figure 7A). The expression levels of these genes were evaluated using real-time PCR and Western blotting (Figure 7B,C). Moreover, we found 16 paired samples show elevated PFDN1 mRNA and protein expression (16/20); 17 paired samples show elevated PFDN2 mRNA and protein expression (17/20); 15 paired samples show elevated VBP1 mRNA and protein expression (15/20); 14 paired samples show elevated PFDN4 mRNA and protein expression (14/20) in our clinical samples. The results were consistent with the findings described previously. In addition, we detected the expression of PFDN1/2/3/4 in the normal liver cell line WRL-68 and five HCC cell lines using real-time PCR and Western blotting. Increased levels of PFDN1/2/3/4 were observed in HCC cell lines compared with the normal liver cell line (Figure 7D,E). Therefore, PFDN1/2/3/4 could be reliable and new biomarkers for diagnosis of patients with HCC. Further experiments are needed to reveal the potential molecular functions of PFDN1/2/3/4 in HCC.

## Discussion

Although significant achievements in the treatment methods and diagnosis of HCC have been reported, the 5-year survival of patients is far from satisfactory (Iguchi et al., 2015; Yang et al., 2016). We must further explore reliable and new biomarkers for HCC. Our study was the first to comprehensively elucidate the prognostic value of the PFDN family in HCC using the TCGA database. Although an increased expression of the PFDN family was detected in the TCGA database, only PFDN1–4 showed prognostic value in patients with HCC. We also identified increased mRNA and protein expression of PFDN1–4 in our clinical samples. These findings might enhance the accuracy of diagnosis and provide new targets for precision therapy for patients with HCC.

PFDNs, containing six different subunits (PFDN1–6), act as a co-chaperone of the chaperonin CCT in the folding of cytoskeletal proteins. In addition to its classical function, the PFDN family also participates in maintaining cytoplasmic proteostasis. Recently, PFDNs have been shown to be involved in nuclear functions, including binding the transcription start sites of genes, recruiting transcriptional corepressors, and regulating co-transcriptional splicing.

Previous studies have illustrated that PFDNs participate in various diseases. In mucinous colorectal adenocarcinoma (MAC), PFDN1 is a direct target of filamin A interacting protein 1-like (FILIP1L) and is degraded by the proteasome. PFDN1 induces neoplastic characteristics in aggressive aneuploid MAC (Kwon et al., 2021). PFDN1 also functions as an indicator of poor prognosis in patients with non-small cell lung cancer (Peñate et al., 2020). In our study, significantly increased expression of the PFDN1 was found in HCC tissues. In addition, higher expression of the PFDN1 potentially predicts a shorter OS and PFI for patients with HCC. PFDN1 also has a close relationship with worse T stage, higher AFP level, and histological grade. We performed GSEA to analyze the potential function of PFDN1 in HCC. The interleukin-related signaling pathways, Rho GTPase pathways, and the PI3K–AKT pathway were significantly enriched in the PFDN1-high group. A PFDN1-related nomogram was constructed with PFDN1 expression and other clinical variables (T stage, N stage, and pathological stage). The C-index of the PFDN1-related Cox model for predicting overall survival was 0.647 (95% CI: 0.618–0.677).

PFDN2, a subunit of the PFDN complex, has been reported to regulate the cytoskeleton. PFDN2 was found to be associated with type 2 diabetes (Chang et al., 2017) and predicts poor prognosis for patients with metastatic urothelial carcinoma (Riester et al., 2014). In this study, an increased mRNA level of PFDN2 was observed to be significantly associated with the T stage, AFP level, histological grade, and vascular invasion. Patients with HCC in the PFDN2-high group experienced shorter OS and DSS. GSEA revealed that GPCR ligand binding, DNA repair, mitotic G2-M phase, and cell cycle checkpoint were significantly correlated with PFDN2-high phenotype. Therefore, PFDN2 might perform its function in the nucleus. The C-index of the nomogram containing PFDN2 and clinical features was 0.643 (95% CI: 0.600–0.699).

PFDN3, also known as VBP1, might play opposite roles in different cancers. In addition to its canonical role in promoting newly synthesized protein folding, VBP1 is related to proteasome- and autophagy-mediated protein degradation. VBP1 binds HBx and induces NF-κB activation in HCC (Kim et al., 2008). VBP1 also predicts poor prognosis for patients with gastric cancer and non-small cell lung cancer (Peñate et al., 2020; Yesseyeva et al., 2020). However, VBP1 was reported to suppress the EMT in cancer (Kim et al., 2018). In this study, we documented that higher expression of VBP1 predicted poorer prognosis (OS and PFI) for patients with HCC and was significantly associated with an advanced T stage and pathological stage. Functional analysis, which included GPCR ligand binding, Rho GTPase signaling pathway, PI3K–AKT signaling pathway, and DNA repair, was enriched in the VBP1-high group. Hence, we speculated that VBP1 might promote HCC progression through its functions in the cytoplasm and nucleus. The C-index of the nomogram combining VBP1 and other clinical features was 0.623 (95% CI: 0.588–0.657).

PFDN4 might promote tumor progression through multiple pathways. PFDN4, when combined with SHC4 and CHORDC1, regulates extracellular vesicle secretion in prostate cancer (Urabe et al., 2020). The correlations between PFDN4 expression and clinical parameters were similar to those of PFDN1. Higher PFDN4 expression was related to a poorer prognosis (OS, PFI, and DSS) of patients with HCC. GSEA results indicated that GPCR ligand binding, Rho GTPase signaling pathways, and processing of capped introns containing pre-mRNA were significantly associated with PFDN4 alteration. Thus, PFDN4 might promote the development of HCC by regulating the splicing of pre-mRNA. The deeper mechanism of PFDN4 in HCC requires further investigation. The C-index of the PFDN4 nomogram was 0.650 (95% CI: 0.617–0.682).

Recently, the focus of researchers had shifted to tumor-infiltrating immune cells, which might influence the progression and metastasis of cancer. As shown in our study, PFDN1-4 expression levels were positively associated with Th2 and Tfh cell infiltration. Previous studies had illustrated that Th2 cells and Tfh cells might contribute to development of cancer. Therefore, these results implied that PFDN1-4 promoted the tumorigenesis of HCC by modulating immune cell infiltration.

We employed the STRING database to identify related genes and subsequently analyze these genes using Cytoscape to explore the molecular functions of PFDN1-4. GO and KEGG analyses showed that in addition to their canonical functions, such as unfolded protein binding and microtubule and chaperon complexes, PFDN1-4 participated in nuclear functions. These functions included regulation of protein localization to the nucleus and chromosome. These findings were consistent with those of some recent studies that PFDNs participate in folding transcriptional regulators and regulating pre-mRNA splicing. These mechanisms require further study and might help us identify new therapeutic targets in HCC.

Although PFDN5 and PFDN6 showed differential expression in HCC tissues compared with adjacent tissues, no connections were observed between the expression of these genes and prognostic value. Perhaps, we should further explore the prognostic values of PFDN5 and PFDN6 in our own samples.

We must admit that some limitations existed in the present study. The detailed mechanisms by which PFDN1/2/3/4 modulated HCC progression, immune cell infiltration, and signaling pathway activation require further investigation. Moreover, the prognostic value of PFDN1–4 requires further validation and amendment in a larger clinical cohort.

In conclusion, by performing a bioinformatics analysis, we clarified that PFDN1–4 possess significant potential as novel therapeutic targets and prognostic biomarkers in HCC. In-depth biological experiments and larger-scale clinical samples are needed to confirm our conclusions.

## Conclusion

In conclusion, this study first reported the PFDN family in HCC. Our study demonstrated that upregulated expression of the PFDN family in HCC, while only PFDN1/2/3/4 showed prognostic value in the TCGA database. The expression of PFDN1/2/3/4 was significantly associated with advanced clinical parameters of patients with HCC. PFDN1/2/3/4 also regulated Th2 and Tfh cell infiltration. Alteration of PFDN1/2/3/4 was related to cell cycle checkpoints, G2-M phases, Rho GTPase activation, and GPCR ligand binding. Our clinical samples further confirmed the increased expression of PFDN1/2/3/4 in HCC tissues compared with adjacent tissues. These results implied that PFDN1/2/3/4 could be effective biomarkers for patients with HCC. The potential biological mechanisms of PFDN family in HCC need further exploration.

## Data Availability

The datasets presented in this study can be found in online repositories. The names of the repository/repositories and accession number(s) can be found in the article/Supplementary Material.

## Supplementary material

Supplementary Figure S1 ROC curves of the PFDN family in HCC. The ROC curves of PFDN1–6 (A–F) in HCC.

Supplementary Figure S2 Scatter plots indicate the correlations between PFDN1/2/3/4 expression and (A) Th2 cells and (B) TFH cells.

Supplementary Table S1 TCGA HCC patient characteristics.

Supplementary Figure S2 Primer information

Supplementary Figure S3
Antibody information

Supplementary Table S4 DEGs of PFDN1.

Supplementary Table S5 DEGs of PFDN2.

Supplementary Table S6 DEGs of PFDN3.

Supplementary Table S7 DEGs of PFDN4.

Supplementary Table S8 PFDN1-related GSEA analysis.

Supplementary Table S9 PFDN2-related GSEA analysis.

Supplementary Table S10 PFDN3-related GSEA analysis.

Supplementary Table S11 PFDN4-related GSEA analysis.

Supplementary Table S12 GO and KEGG analysis.

## References

[B1] Bindea G. Mlecnik B. Tosolini M. Kirilovsky A. Waldner M. Obenauf A. C. (2013). Spatiotemporal dynamics of intratumoral immune cells reveal the immune landscape in human cancer. Immunity 39 (4), 782–795. 10.1016/j.immuni.2013.10.003 24138885

[B2] Boulon S. Bertrand E. Pradet-Balade B. (2012). Hsp90 and the R2tp Co-chaperone complex: Building multi-protein machineries essential for cell growth and gene expression. RNA Biol. 9 (2), 148–154. 10.4161/rna.18494 22418846

[B3] Chang D. C. Piaggi P. Hanson R. L. Knowler W. C. Bogardus C. Krakoff J. (2017). Autoantibodies against Pfdn2 are associated with an increased risk of type 2 diabetes: A case-control study. Diabetes. Metab. Res. Rev. 33 (8), e2922. 10.1002/dmrr.2922 PMC6417877 28731290

[B4] Craig A. J. von Felden J. Garcia-Lezana T. Sarcognato S. Villanueva A. (2020). Tumour evolution in hepatocellular carcinoma. Nat. Rev. Gastroenterol. Hepatol. 17 (3), 139–152. 10.1038/s41575-019-0229-4 31792430

[B5] Crotty S. T. (2019). T follicular helper cell biology: A decade of discovery and diseases. Immunity 50 (5), 1132–1148. 10.1016/j.immuni.2019.04.011 31117010 PMC6532429

[B6] De Monte L. Reni M. Tassi E. Clavenna D. Papa I. Recalde H. (2011). Intratumor T helper type 2 cell infiltrate correlates with cancer-associated fibroblast thymic stromal lymphopoietin production and reduced survival in pancreatic cancer. J. Exp. Med. 208 (3), 469–478. 10.1084/jem.20101876 21339327 PMC3058573

[B7] Dehghan-Nayeri N. Rezaei-Tavirani M. Omrani M. D. Gharehbaghian A. Goudarzi Pour K. Eshghi P. (2017). Identification of potential predictive markers of dexamethasone resistance in childhood acute lymphoblastic leukemia. J. Cell Commun. Signal. 11 (2), 137–145. 10.1007/s12079-016-0357-3 27778231 PMC5440342

[B8] Esteve-Bruna D. Carrasco-López C. Blanco-Touriñán N. Iserte J. Calleja-Cabrera J. Perea-Resa C. (2020). Prefoldins contribute to maintaining the levels of the spliceosome lsm2-8 complex through Hsp90 in arabidopsis. Nucleic Acids Res. 48 (11), 6280–6293. 10.1093/nar/gkaa354 32396196 PMC7293050

[B9] Fan S. Chen Y. Jiang Y. Hu K. Li C. (2020). Prefoldin subunit Mm1 promotes cell migration via facilitating filopodia formation. Biochem. Biophys. Res. Commun. 533 (3), 613–619. 10.1016/j.bbrc.2020.09.063 32981679

[B10] Fleming C. Morrissey S. Cai Y. Yan J. (2017). Γδ T cells: Unexpected regulators of cancer development and progression. Trends Cancer 3 (8), 561–570. 10.1016/j.trecan.2017.06.003 28780933 PMC5551453

[B11] Geier A. Gartung C. Dietrich C. G. (2002). Hepatitis B E antigen and the risk of hepatocellular carcinoma. N. Engl. J. Med. 347 (21), 1721–1722. 10.1056/nejm200211213472119 12444194

[B12] Hänzelmann S. Castelo R. Guinney J. (2013). Gsva: Gene set variation analysis for microarray and rna-seq data. BMC Bioinforma. 14, 7. 10.1186/1471-2105-14-7 PMC3618321 23323831

[B13] Houry W. A. Bertrand E. Coulombe B. (2018). The paqosome, an r2tp-based chaperone for quaternary structure formation. Trends biochem. Sci. 43 (1), 4–9. 10.1016/j.tibs.2017.11.001 29203338

[B14] Iguchi T. Shirabe K. Aishima S. Wang H. Fujita N. Ninomiya M. (2015). New pathologic stratification of microvascular invasion in hepatocellular carcinoma: Predicting prognosis after living-donor liver transplantation. Transplantation 99 (6), 1236–1242. 10.1097/tp.0000000000000489 25427164

[B15] Ji J. Wang X. W. (2012). Clinical implications of cancer stem cell biology in hepatocellular carcinoma. Semin. Oncol. 39 (4), 461–472. 10.1053/j.seminoncol.2012.05.011 22846863 PMC3409471

[B16] Kim J. A. Choi D. K. Min J. S. Kang I. Kim J. C. Kim S. (2018). Vbp1 represses cancer metastasis by enhancing hif-1α degradation induced by pvhl. FEBS J. 285 (1), 115–126. 10.1111/febs.14322 29121446

[B17] Kim S. Y. Kim J. C. Kim J. K. Kim H. J. Lee H. M. Choi M. S. (2008). Hepatitis B virus X protein enhances nfkappab activity through cooperating with Vbp1. BMB Rep. 41 (2), 158–163. 10.5483/bmbrep.2008.41.2.158 18315953

[B18] Kimura Y. Nagao A. Fujioka Y. Satou A. Taira T. Iguchi-Ariga S. M. (2007). Mm-1 facilitates degradation of C-myc by recruiting proteasome and a novel ubiquitin E3 ligase. Int. J. Oncol. 31 (4), 829–836. 10.3892/ijo.31.4.829 17786314

[B19] Kwon M. Rubio G. Nolan N. Auteri P. Volmar J. A. Adem A. (2021). Filip1l loss is a driver of aggressive mucinous colorectal adenocarcinoma and mediates cytokinesis defects through Pfdn1. Cancer Res. 81, 5523–5539. 10.1158/0008-5472.Can-21-0897 34417201 PMC8563430

[B20] Llovet J. M. Villanueva A. Lachenmayer A. Finn R. S. (2015). Advances in targeted therapies for hepatocellular carcinoma in the genomic era. Nat. Rev. Clin. Oncol. 12 (8), 436. 10.1038/nrclinonc.2015.121 26099984

[B21] Miyoshi N. Ishii H. Mimori K. Nishida N. Tokuoka M. Akita H. (2010). Abnormal expression of Pfdn4 in colorectal cancer: A novel marker for prognosis. Ann. Surg. Oncol. 17 (11), 3030–3036. 10.1245/s10434-010-1138-5 20552408

[B22] Mo S. J. Zhao H. C. Tian Y. Z. Zhao H. L. (2020). The role of prefoldin and its subunits in tumors and their application prospects in nanomedicine. Cancer Manag. Res. 12, 8847–8856. 10.2147/cmar.S270237 33061580 PMC7520118

[B23] Mori K. Maeda Y. Kitaura H. Taira T. Iguchi-Ariga S. M. Ariga H. (1998). Mm-1, a novel C-Myc-Associating protein that represses transcriptional activity of C-myc. J. Biol. Chem. 273 (45), 29794–29800. 10.1074/jbc.273.45.29794 9792694

[B24] Payán-Bravo L. Fontalva S. Peñate X. Cases I. Guerrero-Martínez J. A. Pareja-Sánchez Y. (2021). Human prefoldin modulates Co-transcriptional pre-mrna splicing. Nucleic Acids Res. 49 (11), 6267–6280. 10.1093/nar/gkab446 34096575 PMC8216451

[B25] Payán-Bravo L. Peñate X. Chávez S. (2018). Functional contributions of prefoldin to gene expression. Adv. Exp. Med. Biol. 1106, 1–10. 10.1007/978-3-030-00737-9_1 30484149

[B26] Peñate X. Praena-Fernández J. M. Romero Pareja P. Enguix-Riego M. D. V. Payán-Bravo L. Vieites B. (2020). Overexpression of canonical prefoldin associates with the risk of mortality and metastasis in non-small cell lung cancer. Cancers (Basel) 12 (4), E1052. 10.3390/cancers12041052 PMC7225921 32344577

[B27] Riester M. Werner L. Bellmunt J. Selvarajah S. Guancial E. A. Weir B. A. (2014). Integrative analysis of 1q23.3 copy-number gain in metastatic urothelial carcinoma. Clin. Cancer Res. 20 (7), 1873–1883. 10.1158/1078-0432.Ccr-13-0759 24486590 PMC3975677

[B28] Roessler S. Jia H. L. Budhu A. Forgues M. Ye Q. H. Lee J. S. (2010). A unique metastasis gene signature enables prediction of tumor relapse in early-stage hepatocellular carcinoma patients. Cancer Res. 70 (24), 10202–10212. 10.1158/0008-5472.Can-10-2607 21159642 PMC3064515

[B29] Shannon P. Markiel A. Ozier O. Baliga N. S. Wang J. T. Ramage D. (2003). Cytoscape: A software environment for integrated models of biomolecular interaction networks. Genome Res. 13 (11), 2498–2504. 10.1101/gr.1239303 14597658 PMC403769

[B30] Szklarczyk D. Gable A. L. Lyon D. Junge A. Wyder S. Huerta-Cepas J. (2019). String V11: Protein-protein association networks with increased coverage, supporting functional discovery in genome-wide experimental datasets. Nucleic Acids Res. 47 (D1), D607–D613. 10.1093/nar/gky1131 30476243 PMC6323986

[B31] Tsao M. L. Chao C. H. Yeh C. T. (2006). Interaction of hepatitis C virus F protein with prefoldin 2 perturbs tubulin cytoskeleton organization. Biochem. Biophys. Res. Commun. 348 (1), 271–277. 10.1016/j.bbrc.2006.07.062 16876117

[B32] Urabe F. Kosaka N. Sawa Y. Yamamoto Y. Ito K. Yamamoto T. (2020). Mir-26a regulates extracellular vesicle secretion from prostate cancer cells via targeting Shc4, Pfdn4, and Chordc1. Sci. Adv. 6 (18), eaay3051. 10.1126/sciadv.aay3051 32494663 PMC7190312

[B33] Vainberg I. E. Lewis S. A. Rommelaere H. Ampe C. Vandekerckhove J. Klein H. L. (1998). Prefoldin, a chaperone that delivers unfolded proteins to cytosolic chaperonin. Cell 93 (5), 863–873. 10.1016/s0092-8674(00)81446-4 9630229

[B34] Villanueva A. (2019). Hepatocellular carcinoma. N. Engl. J. Med. 380 (15), 1450–1462. 10.1056/NEJMra1713263 30970190

[B35] Wang C. Dong L. Li X. Li Y. Zhang B. Wu H. (2021). The pgc1α/nrf1-mpc1 Axis suppresses tumor progression and enhances the sensitivity to sorafenib/doxorubicin treatment in hepatocellular carcinoma. Free Radic. Biol. Med. 163, 141–152. 10.1016/j.freeradbiomed.2020.11.035 33276082

[B36] Wang D. Shi W. Tang Y. Liu Y. He K. Hu Y. (2017). Prefoldin 1 promotes emt and lung cancer progression by suppressing cyclin a expression. Oncogene 36 (7), 885–898. 10.1038/onc.2016.257 27694898 PMC5318667

[B37] Wang P. Zhao J. Yang X. Guan S. Feng H. Han D. (2015). Pfdn1, an indicator for colorectal cancer prognosis, enhances tumor cell proliferation and motility through cytoskeletal reorganization. Med. Oncol. 32 (12), 264. 10.1007/s12032-015-0710-z 26553318

[B38] Yang S. L. Liu L. P. Sun Y. F. Yang X. R. Fan J. Ren J. W. (2016). Distinguished prognosis after hepatectomy of hbv-related hepatocellular carcinoma with or without cirrhosis: A long-term follow-up analysis. J. Gastroenterol. 51 (7), 722–732. 10.1007/s00535-015-1146-0 26607653

[B39] Yesseyeva G. Aikemu B. Hong H. Yu C. Dong F. Sun J. (2020). Prefoldin subunits (Pfdn1-6) serve as poor prognostic markers in gastric cancer. Biosci. Rep. 40 (2), BSR20192712. 10.1042/bsr20192712 31957800 PMC7024841

[B40] Yu G. Wang L. G. Han Y. He Q. Y. (2012). Clusterprofiler: An R package for comparing biological themes among gene clusters. Omics a J. Integr. Biol. 16 (5), 284–287. 10.1089/omi.2011.0118 PMC3339379 22455463

