# Supplementary material for: Comprehensive analysis of the prognostic value and functions of prefoldins in hepatocellular carcinoma
Source: Front Mol Biosci. 2022 Nov 11;9:957001. doi: 10.3389/fmolb.2022.957001 (PMC9691963; doi:10.3389/fmolb.2022.957001)
Supplement: Supplementary file 1 [file Table1.DOCX]

| Characteristic | levels | Overall |
| --- | --- | --- |
| n |  | 374 |
| T stage, n (%) | T1 | 183 (49.3%) |
|  | T2 | 95 (25.6%) |
|  | T3 | 80 (21.6%) |
|  | T4 | 13 (3.5%) |
| N stage, n (%) | N0 | 254 (98.4%) |
|  | N1 | 4 (1.6%) |
| M stage, n (%) | M0 | 268 (98.5%) |
|  | M1 | 4 (1.5%) |
| Pathologic stage, n (%) | Stage I | 173 (49.4%) |
|  | Stage II | 87 (24.9%) |
|  | Stage III | 85 (24.3%) |
|  | Stage IV | 5 (1.4%) |
| Tumor status, n (%) | Tumor free | 202 (56.9%) |
|  | With tumor | 153 (43.1%) |
| Gender, n (%) | Female | 121 (32.4%) |
|  | Male | 253 (67.6%) |
| Race, n (%) | Asian | 160 (44.2%) |
|  | Black or African American | 17 (4.7%) |
|  | White | 185 (51.1%) |
| Age, n (%) | <=60 | 177 (47.5%) |
|  | >60 | 196 (52.5%) |
| Weight, n (%) | <=70 | 184 (53.2%) |
|  | >70 | 162 (46.8%) |
| Height, n (%) | < 170 | 201 (58.9%) |
|  | >=170 | 140 (41.1%) |
| BMI, n (%) | <=25 | 177 (52.5%) |
|  | >25 | 160 (47.5%) |
| Residual tumor, n (%) | R0 | 327 (94.8%) |
|  | R1 | 17 (4.9%) |
|  | R2 | 1 (0.3%) |
| Histologic grade, n (%) | G1 | 55 (14.9%) |
|  | G2 | 178 (48.2%) |
|  | G3 | 124 (33.6%) |
|  | G4 | 12 (3.3%) |
| Adjacent hepatic tissue inflammation, n (%) | None | 118 (49.8%) |
|  | Mild | 101 (42.6%) |
|  | Severe | 18 (7.6%) |
| AFP(ng/ml), n (%) | <=400 | 215 (76.8%) |
|  | >400 | 65 (23.2%) |
| Albumin(g/dl), n (%) | <3.5 | 69 (23%) |
|  | >=3.5 | 231 (77%) |
| Prothrombin time, n (%) | <=4 | 208 (70%) |
|  | >4 | 89 (30%) |
| Child-Pugh grade, n (%) | A | 219 (90.9%) |
|  | B | 21 (8.7%) |
|  | C | 1 (0.4%) |
| Fibrosis ishak score, n (%) | 0 | 75 (34.9%) |
|  | 1/2 | 31 (14.4%) |
|  | 3/4 | 28 (13%) |
|  | 5/6 | 81 (37.7%) |
| Vascular invasion, n (%) | No | 208 (65.4%) |
|  | Yes | 110 (34.6%) |
| Age, median (IQR) |  | 61 (52, 69) |
