# Supplementary figures and images for: Comprehensive analysis of the prognostic value and functions of prefoldins in hepatocellular carcinoma
Source: Front Mol Biosci. 2022 Nov 11;9:957001. doi: 10.3389/fmolb.2022.957001 (PMC9691963; doi:10.3389/fmolb.2022.957001)

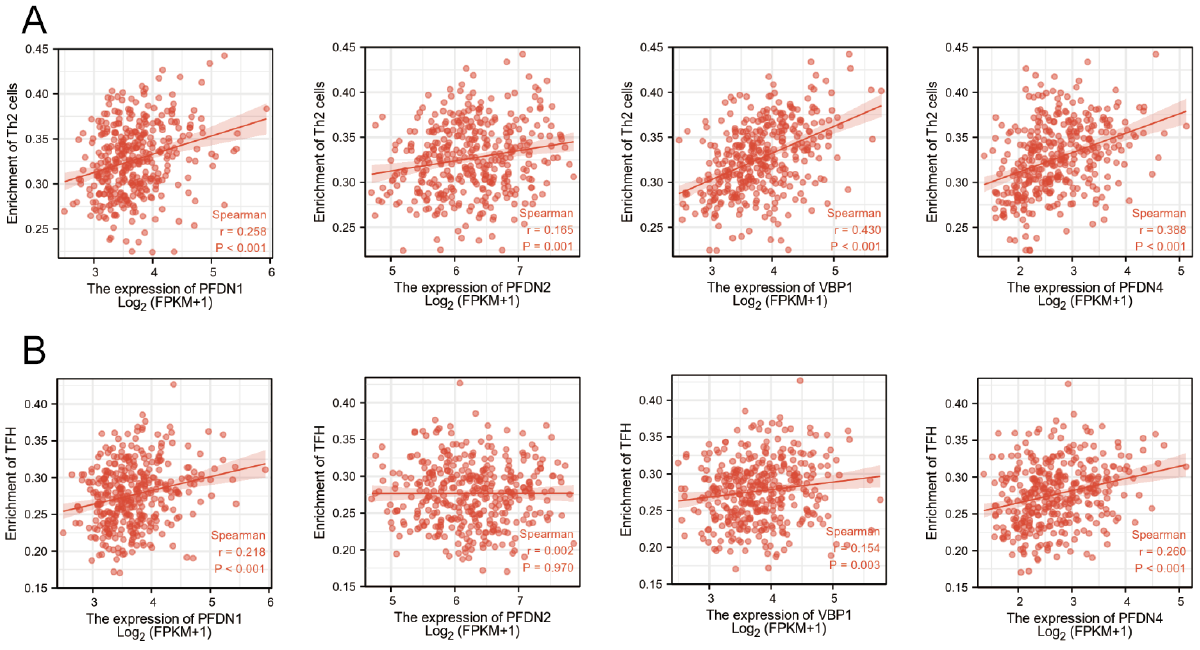

Supplement: Supplementary file 3 [file Image2.TIF]

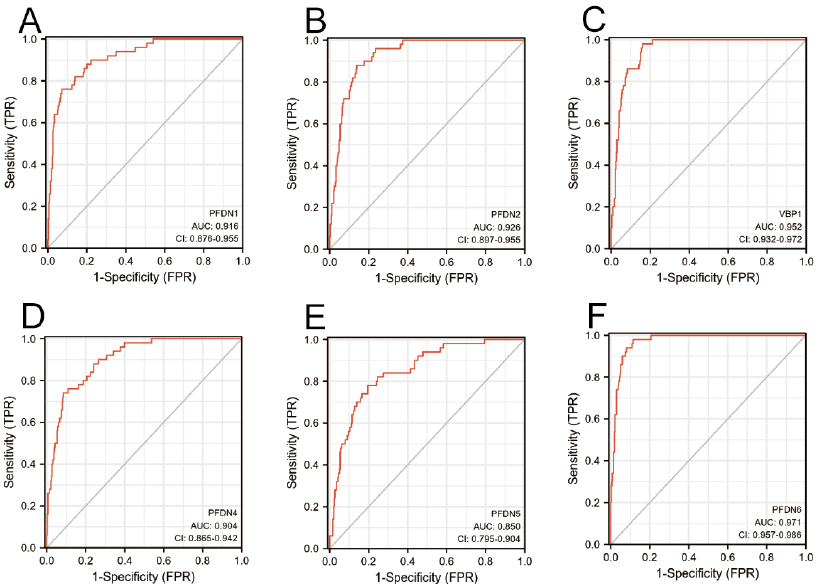

Supplement: Supplementary file 4 [file Image1.TIF]
