# Supplementary material for: Comprehensive analysis of the prognostic value and functions of prefoldins in hepatocellular carcinoma
Source: Front Mol Biosci. 2022 Nov 11;9:957001. doi: 10.3389/fmolb.2022.957001 (PMC9691963; doi:10.3389/fmolb.2022.957001)
Supplement: Supplementary file 6 [file Table2.DOCX]

**Supplementary table 2 Primer information**

| Gene Name | Sequence (5′-3′) |
| --- | --- |
| PFDN1 | F: CAGAACGAAAAAGCATGCAC |
|  | R: CTTCGTGCCATCAGCATCT |
| PFDN2 | F: ATGAGCACAGCCTAGTGATCG |
|  | R: ACTCCTCCAACCATGCGGTA |
| VBP1 | F:AGTCCACCAACTCAATGGAGA |
|  | R: CAAGCATTACATTAGCCCCCAA |
| PFDN4 | F: AGTGGAATCAATTCAGCGAGTG |
|  | R: GCTTCAAGGTTTATGTTGCTCCC |
| GAPDH | F: GAAATCCCATCACCATCTTCCAGG |
|  | R: CAGTAGAGGCAGGGATGATGTTC |
