# Supplementary material for: Comprehensive analysis of the prognostic value and functions of prefoldins in hepatocellular carcinoma
Source: Front Mol Biosci. 2022 Nov 11;9:957001. doi: 10.3389/fmolb.2022.957001 (PMC9691963; doi:10.3389/fmolb.2022.957001)
Supplement: Supplementary file 9 [file Table3.DOCX]

**Supplementary Table 3 Antibody information**

| Antibody | Host | Product number | Company | Dilution |
| --- | --- | --- | --- | --- |
| PFDN1 | Rabbit | ab151708 | Abcam | 1:1000(WB)  1:100(IHC) |
| PFDN2 | Rabbit | 13053-1-AP | Proteintech | 1:1000(WB)  1:200(IHC) |
| VBP1 | Rabbit | 14345-1-AP | Proteintech | 1:1000(WB)  1:200(IHC) |
| PFDN4 | Rabbit | 16045-1-AP | Proteintech | 1:1000(WB)  1:200(IHC) |
| GAPDH | Mouse | 60004-1-Ig | Proteintech | 1:10000(WB) |
